# Transforming Aluminum-Ion Batteries with Recyclable Solid-State Electrolytes

**DOI:** 10.1021/acscentsci.5c00224

**Published:** 2025-02-18

**Authors:** Zhitong Xiao, Quanquan Pang

**Affiliations:** Beijing Key Laboratory for Theory and Technology of Advanced Battery Materials, School of Materials Science and Engineering, Peking University, Beijing 100871, China

**Figure 1:**
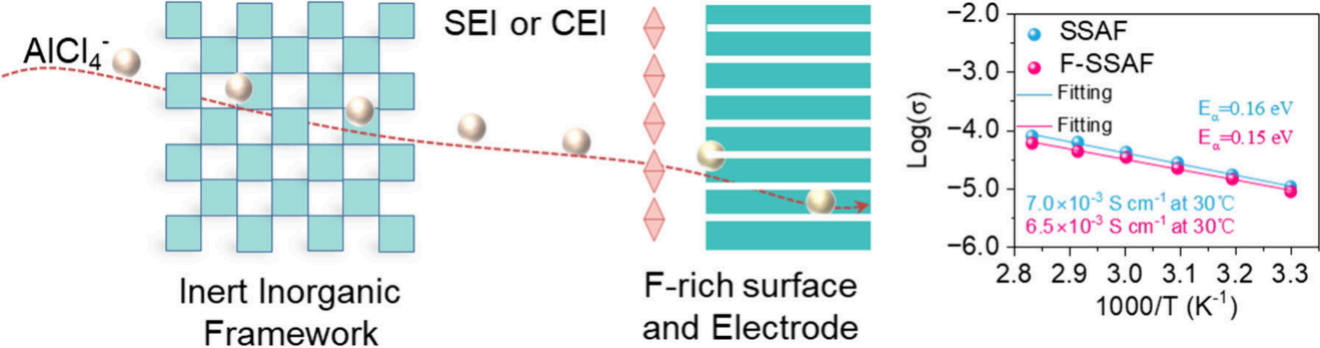
Schematic diagram of electrolyte framework and interface design with high ionic conductivity. SEI, solid electrolyte interphase. CEI, cathode electrolyte interphase. Reproduced with permission from ref (1). Copyright 2024 American Chemical Society.

Aluminum-ion batteries (AIBs) represent a promising candidate for large-scale energy storage systems (ESSs), showcasing notable benefits such as superior safety, low materials cost, and operational versatility across a broad temperature spectrum.^1−4^ However, their application has been constrained by challenges including the corrosive nature and poor reaction kinetics of conventional chloroaluminate-based ionic liquid electrolytes (ILs).^5,6^ Efforts have been made on high-energy-density aluminum–sulfur batteries employing inorganic chloride molten electrolytes, which represent high safety, cost-effectiveness, and fast-charging capabilities.^7^ Researchers are also exploring batteries with room-temperature molten salt electrolytes, encapsulated within polymers or metal–organic frameworks.^8−10^ These efforts address critical issues related to safety and performance, yet challenges such as high production costs and scalability persist as significant hurdles. In the current issue of *ACS Central Science*, Jiao, Wang, and co-workers introduce a recyclable solid-state electrolyte (denoted as F-SSAF),^1^ which overcomes these limitations and improves the lifespan and safety of AIBs (Figure 1).

The F-SSAF electrolyte features a design that combines an inert aluminum fluoride (AlF_3_) inorganic framework, 1-ethyl-3-methylimidazolium chloride with AlCl_3_ (EMIC-AlCl_3_), as the electrolyte, and FEC@EMIC-AlCl_3_ (FIL) as an interface additive. This design embodies several significant advancements. First, the AlF_3_ framework functions as a solid diluent, enhancing the dissociation of Al_2_Cl_7_^–^ into AlCl_4_^–^, thereby facilitating active ion migration and mitigating aluminum anode corrosion. Second, the F-SSAF design reduces the dependency on expensive EMIC-AlCl_3_ while enhancing resistance to moisture, rendering the electrolyte more cost-effective and environmentally sustainable. Additionally, the FIL interface additive assists in the formation of F-rich solid electrolyte interphases and cathode electrolyte interphases. These interphases ensure uniform aluminum deposition and prevent dendrite growth, addressing a critical challenge in extending battery lifespan. The system exhibits outstanding performance, facilitating stable aluminum deposition and dissolution for up to 4,000 h in symmetric cells and over 10,000 cycles in full cells while maintaining a Coulombic efficiency exceeding 99%. The recyclability of the AlF_3_ framework provides another key advantage by reducing the environmental impact and production costs. With an 80% recycling rate achieved in this study, the findings highlight the potential for integrating sustainable practices into the advancement of next-generation batteries. Future innovations may potentially facilitate near-complete recyclability of AlF_3_, thereby improving cost efficiency. These advancements not only enhance the industrial viability of AIBs but also contribute to global sustainability efforts.

This research represents a significant advancement in addressing the growing need for sustainable and efficient ESSs. By addressing critical limitations of conventional ILs, the F-SSAF electrolyte opens up new avenues for the large-scale application of AIBs in renewable energy storage, electric grids, and industrial power systems. Future research can explore the potential of other inorganic frameworks, optimizing their structure and morphology for improved performance. Advanced materials design methodologies, such as high-throughput screening and machine learning algorithms, could accelerate the discovery and customization of novel frameworks with superior properties. These techniques may enable further advancements in ion transport, interface stability, and overall system efficiency.

The work from Jiao, Wang, and co-workers not only advances the state-of-the-art AIBs but also paves the way for more sustainable and scalable battery technologies. As ESSs become increasingly important in global sustainability efforts, such innovations will undoubtedly play a pivotal role in shaping the next generation of high-performance batteries. By addressing key challenges in electrolyte design, this study provides a compelling blueprint for overcoming the trade-offs among performance, safety, and cost in energy storage solutions.
